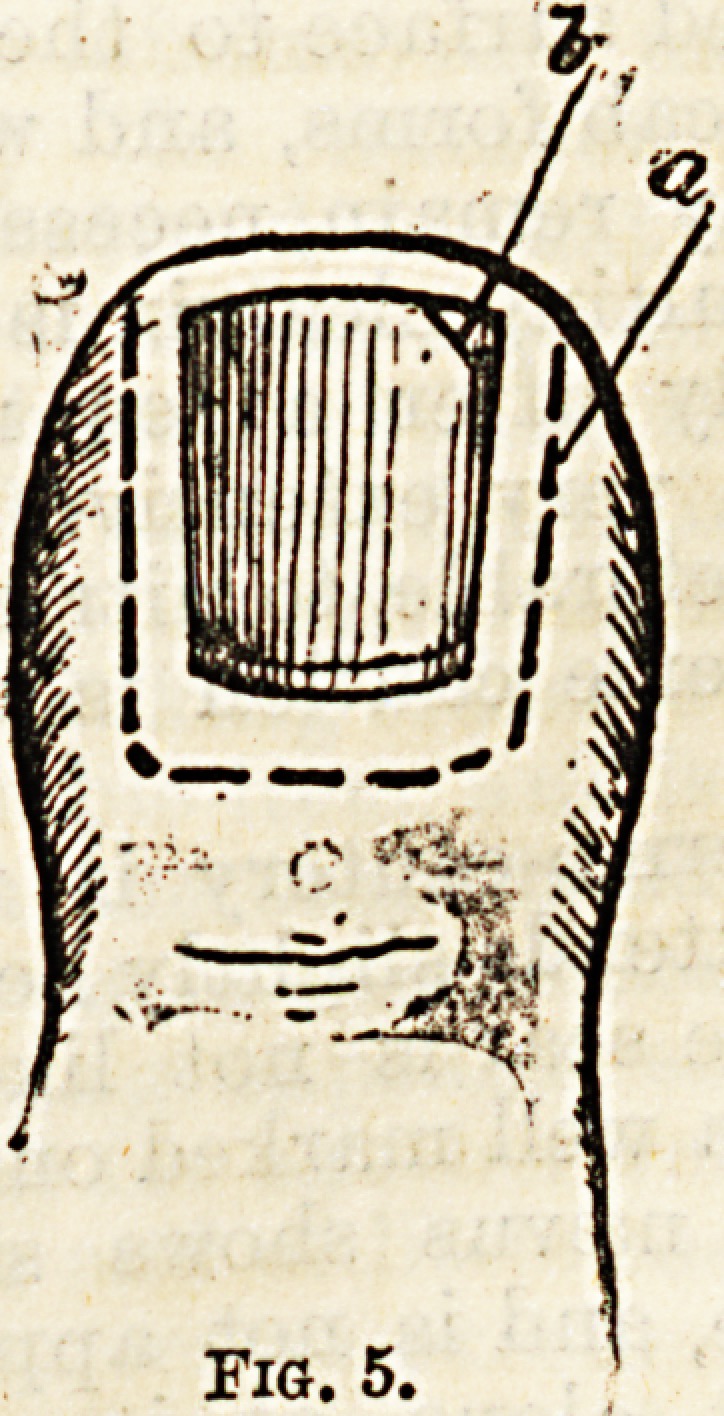# Ingrowing Toe-nail

**Published:** 1893-10-07

**Authors:** G. Munro Smith

**Affiliations:** Senior Assistant Surgeon to the Bristol Royal Infirmary


### The Hospital Clinic.

[*The Editor will be glad to receive offers of co-operation and contributions from members of the profession. All letters should be addressed to* The Editor, The Lodge, Porchester Square, London, W.]

### INGROWING TOE-NAIL.

By G. Munro Smith, M.R.C.S., L.R.C.P.Lond., Senior Assistant Surgeon to the Bristol Royal Infirmary.

### II.—Treatment.

The treatment of this affection may be preventive, palliative, or radical.

Prevention consists chiefly in avoiding undue pressure from boots and shoes. Pointed toes, unyielding thick upper leathers, or thin soles which cause the nails to be pressed against the end of the boot, on flexion of the foot—one or other of these is to blame in nearly every case. Injudicious cutting of the nail is often mentioned as a cause. The cut should be straight across, not obliquely downwards at the edges (as shown at *b* in Fig. 5). This probably, however, has very little to do with it; and the fact that the disease is so generally on the outer side of the toe-nail, shows that cutting down the edge may be disregarded.

The principles of the palliative treatment are (1) to remove the offending portion of nail as freely as possible; (2) to keep it from pressing on the sensitive granulations which have sprung up around it; and (3) to destroy these granulations. In the early stages a great deal can be done by patiently removing all the dead nail and heaped-up epidermis which may have accumulated.

Fig. 1.

Fig. 2.

In order to prevent the nail edge from pressing on its surroundings, a great many plans have been devised. The simplest is to carefully pack cotton wool with a probe, or some fine, blunt instrument, carefully under the rough margin, as seen in Fig. 1, where *a* indicates the plug of wool, *c* the nail, and *b* a piece of strapping, which should be fastened round the toe. This should be done every day, or even twice daily, the parts being washed frequently with some antiseptic lotion, or dusted with boric acid powder. Instead of wool, a small piece of oiled rag or lint may be used. Others recommend a bit of sheet lead or silver inserted under the margin. This is mentioned by Mr. Jacobson as one of Professor Humphrey's methods.

On the same principle various little instruments have been used, one of which (Fig. 2) is copied from Messrs. Tiemann and Co.'s catalogue. It is a short metal spring, which can be adjusted to the end of the nail transversely, the part marked *a* coming to the centre, and the small blunt hooks at either end turning over the sides and so protecting the granulations.

Mr. Agnew advocated the application of a piece of cork cut into the shape depicted in Fig. 3. The hook-like process (*a*) is adjusted over the inflamed granulations, so as to keep them away from the irritating nail. The concavity (*b*) is strapped firmly to the side of the toe. It is claimed that this generally succeeds, but it is not well borne by patients who have to move about, unless (which is unusual) the disease is on the inner side of the great toe, and even then the boots must be decidedly broad.

In order to destroy the granulations some strong application is necessary, supplemented by pressure. Two things seem specially useful, viz., pure chromic acid, and carbolic acid with equal parts of cocaine dissolved in a little glycerine. After one of these is applied a band of strapping should be made to encircle the toe. After the application of a 10 per cent. solution of cocaine the granulation may be scraped away without much pain; but this plan of treatment alone is unsatisfactory.

Fig. 3.

Fig. 4.

Unfortunately, whatever palliative measure be adopted, the case often demands more radical treatment. For these operations an anæsthetic is needful, and ether or chloroform are the best suited. Nitrous oxide is hardly satisfactory, as the parts are exquisitely tender, and a minute or two is required to do the requisite cutting. Most surgeons now only remove a portion of the nail. This is done by slitting down longitudinally with a scissors and wrenching out the diseased part with a forceps; then cutting away the unhealthy lateral fold of skin, &c., in the direction shown in fig 4 (*a—a*). By this a cure is effected, and certainly in many cases a permanent one. But difficulty may arise with the free edge which is left and a further removal may be required.

Another partial operation has been recommended by Mr. William Howard, and consists in the removal of a semi-circular slice of flesh in front and at the sides of the nail; when the edges of this wound are brought together by sutures the inflamed granulations are drawn away from the nail. Sufficient time has not yet elapsed, however, to test the utility of this method.

The complete operation is best done by removing the whole nail in the usual manner, and then cutting round with a scalpel in the direction of the dotted lines in fig. 5. When the tags of skin so loosened have been removed, the vascular bed of the nail should be scraped or sliced off until the papillæ of the corium from which the nail grows has been thoroughly destroyed. Thus, all the parts from which a new and distorted nail might grow are removed; and if effectually carried out, the

Fig. 5.

cure may be said to be complete. If, as rarely happens, the nail should grow again, the matrix has not been sufficiently destroyed.

The success of all the less radical plans of treatment depends in a great measure on the persistence of the various means employed, and on the possibility of the patient's aiding the surgeon by proper attention to the fit of the boot, and by resting the inflamed part.

## Figures and Tables

**Fig. 1. f1:**
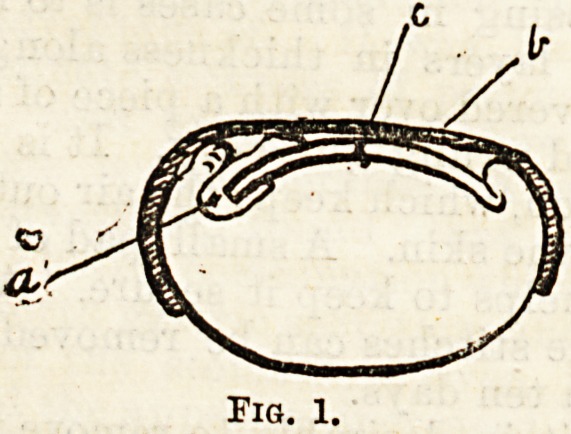


**Fig. 2. f2:**
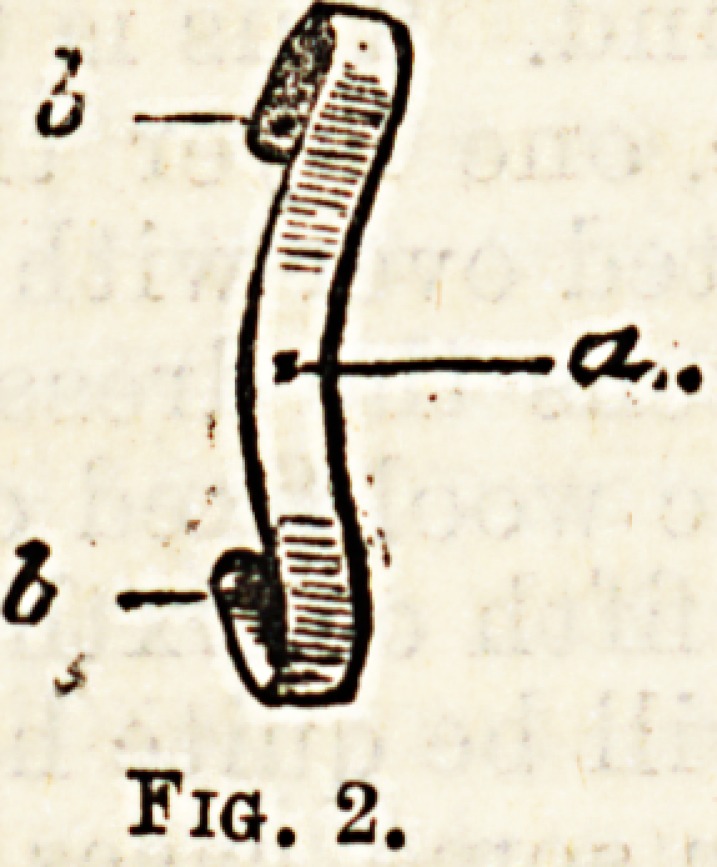


**Fig. 3. f3:**
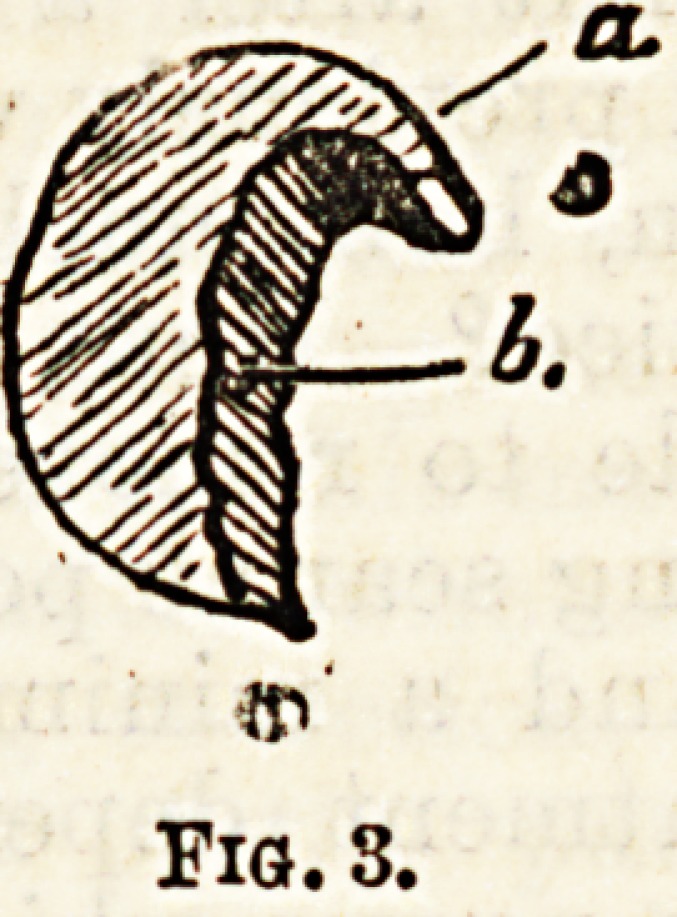


**Fig. 4. f4:**
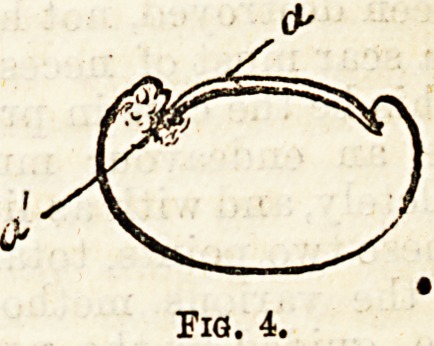


**Fig. 5. f5:**